# Revising the essential package of health services through stakeholder alignment, Somalia

**DOI:** 10.2471/BLT.23.289733

**Published:** 2023-09-28

**Authors:** Mohamed A Jama, Reza Majdzadeh, Teri Reynolds, Ibrahim M Nur, Abdullahi A Ismail, Nur A Mohamud, Andre Griekspoor, Neil Thalagala, John Fogarty, Mamunur SK Malik, Fawziya A Nur

**Affiliations:** a Ministry of Health and Human Services, Federal Government of Somalia, Corso Somalia Street, P.O. Box 22, Shangani, Mogadishu, Somalia.; b School of Health and Social Care, University of Essex, Colchester, England.; c Department of Integrated Health Services, World Health Organization, Geneva, Switzerland.; d Department of Humanitarian and Emergency Services, World Health Organization, Geneva, Switzerland.; e Family Health Bureau, Health Economics Unit, Ministry of Health, Colombo, Sri Lanka.; f Department of Clinical Services and Systems, World Health Organization, Geneva, Switzerland.; g World Health Organization, Country Office, Mogadishu, Somalia.

## Abstract

**Problem:**

The fragmented health sector in Somalia, burdened by financial challenges and an inadequate regulatory system, struggles to provide equitable essential health services to the entire population.

**Approach:**

To revise an essential package of health services that stakeholders could support and that aligned with stakeholders’ financial and technical resources, the federal health ministry invited all key stakeholders in 2020 to participate in the revision process of the essential package. The ministry distributed a concept note to invited stakeholders, describing the scope and purpose of the revision process of the essential package. The note also contained a timeline and the expected contribution of each stakeholder. Stakeholders nominated representatives based on their technical expertise and knowledge of the health sector in Somalia.

**Local setting:**

The health sector in Somalia involves multiple stakeholders, including the health ministry and many development partners. The private sector plays a substantial role in health-care provision. Public spending is an estimated 17% of the total health expenditure.

**Relevant changes:**

After an 18-month revision process, the health ministry and development partners agreed to prioritize high-impact, cost-effective services and use a progressive realization of the package to improve access and coverage. The implementation strategy considers the health system and operational capacity of service providers, particularly in security-compromised areas.

**Lessons learnt:**

The approach showed that inclusivity, collaboration and transparency were of importance for a successful revision of the package. These achievements in consensus-building and priority alignment advance the government’s pursuit of equitable and comprehensive health care for all.

## Résumé

**Problème:**

Le secteur fragmenté de la santé en Somalie, accablé par des problèmes financiers et un système réglementaire inadapté, peine à fournir des services de santé essentiels et équitables à l'ensemble de la population.

**Approche:**

Afin de réviser un programme essentiel de services de santé que les parties prenantes pourraient soutenir et qui correspondrait aux ressources financières et techniques de ces parties prenantes, le ministère fédéral somalien de la Santé a invité en 2020 toutes les parties prenantes clés à participer au processus de révision de ce programme essentiel. Il a distribué aux parties prenantes invitées une note de synthèse décrivant la portée et l'objectif du processus de révision du programme essentiel de services. Cette note contenait également un calendrier et la contribution attendue de chaque partie prenante. Les parties prenantes ont désigné leurs représentants selon leur expertise technique et leur connaissance du secteur de la santé en Somalie.

**Environnement local:**

Le secteur somalien de la santé réunit de multiples parties prenantes, y compris le ministère consacré et de nombreux partenaires de développement. Le secteur privé joue un rôle important dans la fourniture de soins de santé. Les dépenses publiques sont estimées à 17% des dépenses totales de santé.

**Changements significatifs:**

Après un processus de révision de 18 mois, le ministère de la Santé et les partenaires de développement sont convenus d'accorder la priorité aux services à fort impact et rentables et d'appliquer une réalisation progressive du programme afin d'améliorer l'accès aux soins de santé et leur couverture. La stratégie de mise en œuvre tient compte du système de santé et de la capacité opérationnelle des prestataires, en particulier dans les zones où la sécurité est compromise.

**Leçons tirées:**

Cette approche a mis en évidence que l'inclusivité, la collaboration et la transparence étaient essentielles à une révision réussie de l'ensemble du programme. Ces résultats en matière de concertation et d'harmonisation des priorités font progresser le gouvernement dans sa quête de soins de santé équitables et complets pour tous.

## Resumen

**Situación:**

El sector sanitario fragmentado de Somalia, sobrecargado por problemas financieros y un sistema normativo inadecuado, tiene dificultades para prestar servicios sanitarios esenciales equitativos a toda la población.

**Enfoque:**

Para revisar un paquete esencial de servicios sanitarios que las partes interesadas pudieran apoyar y que se ajustara a los recursos financieros y técnicos de las partes interesadas, el Ministerio de Sanidad federal invitó a todas las partes interesadas principales en 2020 a participar en el proceso de revisión del paquete esencial. El ministerio distribuyó una nota conceptual a las partes interesadas invitadas, en la que se describía el alcance y la finalidad del proceso de revisión del paquete esencial. La nota también incluía un calendario y la contribución esperada de cada parte interesada. Las partes interesadas designaron a sus representantes en función de su experiencia técnica y su conocimiento del sector sanitario en Somalia.

**Marco regional:**

Existen varias partes interesadas en el sector sanitario de Somalia, entre ellas el Ministerio de Sanidad y diversos socios para el desarrollo. El sector privado desempeña una función importante en la prestación de asistencia sanitaria. Se calcula que el gasto público representa el 17% del gasto sanitario total.

**Cambios importantes:**

Tras un proceso de revisión de 18 meses, el Ministerio de Sanidad y los socios para el desarrollo acordaron dar prioridad a los servicios de alto impacto y rentables, y emplear una realización progresiva del paquete para mejorar el acceso y la cobertura. La estrategia de ejecución tiene en cuenta el sistema sanitario y la capacidad operativa de los proveedores de servicios, sobre todo en regiones con problemas de seguridad.

**Lecciones aprendidas:**

El enfoque demostró que la inclusión, la colaboración y la transparencia eran importantes para el éxito de la revisión del paquete. Estos logros en materia de creación de consenso y alineación de prioridades hacen avanzar al Gobierno en su empeño por lograr una asistencia sanitaria equitativa e integral para todos.

## ملخص

**المشكلة:**

إن القطاع الصحي المهترئ في الصومال، والمثقل بالتحديات المالية وعدم كفاية النظام التنظيمي، يكافح من أجل توفير خدمات صحية أساسية متكافئة للسكان بالكامل.

**الطريقة:**

بهدف مراجعة حزمة أساسية من الخدمات الصحية التي يمكن لأصحاب المصلحة دعمها، والتي تتماشى مع الموارد المالية والفنية لأصحاب المصلحة، فقد دعت وزارة الصحة الفيدرالية جميع أصحاب المصلحة الرئيسيين في عام 2020 للمشاركة في عملية مراجعة الحزمة الأساسية. ووزعت الوزارة مذكرة للمفاهيم على أصحاب المصلحة المدعوين، تصف نطاق عملية مراجعة الحزمة الأساسية، والغرض منها. وتضمنت المذكرة أيضًا جدولًا زمنيًا، والمساهمة المتوقعة من كل صاحب مصلحة. قام أصحاب المصلحة بانتقاء ممثلين بناءً على خبرتهم الفنية ومعرفتهم بالقطاع الصحي في الصومال.

**المواقع المحلية:**

يضم القطاع الصحي في الصومال العديد من أصحاب المصلحة، بما في ذلك وزارة الصحة والعديد من شركاء التنمية. ويلعب القطاع الخاص دوراً ملموسًا في توفير الرعاية الصحية. بينما يُقدر الإنفاق العام بنحو %17 من إجمالي الإنفاق الصحي.

**التغيّرات ذات الصلة:**

بعد عملية مراجعة استمرت لمدة 18 شهراً، اتفق كل من وزارة الصحة وشركاء التنمية على منح الأولوية للخدمات عالية التأثير وذات الفعالة من حيث التكلفة، واستخدام التحقيق التدريجي للحزمة لتحسين الوصول والتغطية. وتأخذ استراتيجية التنفيذ في الاعتبار النظام الصحي والقدرة التشغيلية لمقدمي الخدمات، وخاصةً في المناطق ذات الخلل الأمني.

**الدروس المستفادة:**

أوضح الأسلوب أن الشمولية والتعاون والشفافية كانت ذات أهمية خاصة في نجاح مراجعة الحزمة. هذه الإنجازات في بناء التوافق وتنسيق الأولويات، تعزز سعي الحكومة لتوفير رعاية صحية متكافئة وشاملة للجميع.

## 摘要

**问题:**

支离破碎的索马里卫生部门深受财务危机和监管体系不完善的拖累，在为所有人民提供公平的基本卫生服务方面举步维艰。

**方法:**

为修正那些利益攸关方可以提供支持并且符合利益攸关方的财务和技术资源的一揽子卫生服务基本方案，2020 年，联邦卫生部邀请所有主要利益攸关方参与一揽子基本方案的修正流程。卫生部给所有受邀利益攸关方发放了一份概念说明，描述了一揽子基本方案修正流程的范围和目的。该说明还包含时间表和每位利益攸关方应作出的贡献。利益攸关方根据其技术专长和对索马里卫生部门的了解提名代表。

**当地状况:**

索马里卫生部门涉及多个利益攸关方，包括卫生部和多个发展合作伙伴。私营部门在提供医疗保健方面发挥着十分重要的作用。公共支出估计占卫生总支出的 17%。

**相关变化:**

经过 18 个月的修正流程之后，卫生部和发展合作伙伴一致同意，将重点放在影响重大、成本效益高的服务上，逐步实施该一揽子方案，以便改善卫生服务的普及情况和覆盖范围。该实施战略将卫生系统和服务方的运营能力纳入考虑范围，尤其是安全受到威胁的地区。

**经验教训:**

该方法表明，包容、协作和透明对于成功修正一揽子方案至关重要。在建立共识和重点事项协调方面取得的成就，推动了政府致力于为所有人提供公平全面的医疗保健的工作。

## Резюме

**Проблема:**

Фрагментированный сектор здравоохранения Сомали, обремененный финансовыми трудностями и неэффективной системой регулирования, не в состоянии обеспечить справедливое предоставление основных медицинских услуг всему населению.

**Подход:**

Для пересмотра базового пакета медицинских услуг, который мог бы быть поддержан заинтересованными сторонами и соответствовал финансовым и техническим ресурсам заинтересованных сторон, Федеральное министерство здравоохранения пригласило все ключевые заинтересованные стороны в 2020 году принять участие в процессе пересмотра данного пакета. Министерство разослало приглашенным заинтересованным сторонам концептуальную записку с описанием масштабов и целей процесса пересмотра базового пакета. В записке также приводились сроки и предполагаемый вклад каждой из заинтересованных сторон. Заинтересованные стороны выдвигали своих представителей с учетом их профессиональной компетентности и знаний в области здравоохранения Сомали.

**Местные условия:**

В секторе здравоохранения Сомали задействовано множество заинтересованных сторон, включая Министерство здравоохранения и многочисленных партнеров по развитию. Существенную роль в оказании медицинских услуг играет частный сектор. Государственные расходы составляют примерно 17% от общих расходов на здравоохранение.

**Осуществленные перемены:**

После 18-месячного процесса пересмотра Министерство здравоохранения и партнеры по развитию пришли к соглашению о приоритетности высокоэффективных, рентабельных услуг и постепенной реализации пакета для улучшения доступа и охвата. Стратегия реализации учитывает состояние системы здравоохранения и оперативный потенциал поставщиков услуг, особенно в районах с ослабленной безопасностью.

**Выводы:**

Этот подход показал, что для успешного пересмотра пакета важны инклюзивность, сотрудничество и прозрачность. Эти достижения в формировании консенсуса и согласовании приоритетов способствуют стремлению правительства к обеспечению справедливого и всеобъемлющего медицинского обслуживания для всех.

## Introduction

A prolonged period of conflict in Somalia has led to a fragile health system in the country and persisting challenges in delivering health-care services. For example, the country’s universal health coverage (UHC) Service Coverage Index score is 27 out of 100, compared with the regional average of 42.5.^1^ The fragmented and often unregulated health services are predominantly provided by the private-for-profit and non-profit sectors. These sectors lack proper coordination or stewardship from the health ministry, which has hindered a successful implementation of the 2009 Essential Package of Health Services.

Recognizing the need for greater coordination among stakeholders to align with national health priorities, the health ministry established an inclusive and participatory coordination structure and a consultative process for revising the country’s essential package of health services. This approach aimed to ensure stakeholder buy-in, leading to successful implementation of the revised health service package. Here we describe how this process was conducted. 

## Local setting

Somalia is experiencing a demographic and epidemiological transition. Maternal, infant and child mortality is decreasing and life expectancy at birth has reached 56.5 years (males: 54.0 years; females: 59.2 years).^2^ Although the maternal mortality ratio has decreased from an estimated 732 deaths per 100 000 live births in 2015 to 692 deaths per 100 000 live births in 2020, it remains high.^3^^,^^4^ Similarly, the infant mortality ratio decreased from 91 deaths per 1000 live births in 2014 to 74 deaths per 1000 live births in 2019, yet it remains higher than in many neighbouring countries.^5^ This transition requires that the Somali health sector is capable of addressing both new and old health problems the population is facing. However, the sector is fragmented and involves multiple stakeholders, including the state health ministries and many development partners. Numerous international and national nongovernmental organizations (NGOs), funded by development partners, provide health services in public facilities. The private sector plays a substantial role in health-care provision, delivering around 60% of health services and 70% of medicines, primarily in urban areas.^6^^,^^7^ The health sector also faces financial challenges. An estimated 17% of total health expenditure is on public spending, while private spending (43%) and donor support (40%) is covering the remaining expenditure.^6^

## Approach

In February 2017, a new government took office, which ushered in a renewed commitment to take leadership in implementing the existing national health sector strategic plan: a roadmap towards achieving UHC through the primary health care approach.

Aligning with Somalia's ninth national health development plan of 2020^8^ and recognizing the need to expand access to essential health services, the government decided to revise the essential package of health services in 2020. The health service package was set as the national framework for organizing, managing and expanding health services. This decision was influenced by improvements in the political and security situation and the anticipation of new funding opportunities from the World Bank.

**Fig. 1:**
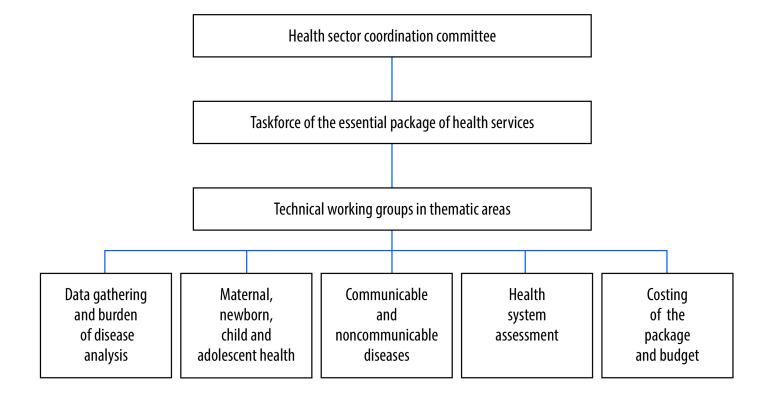
Organizational chart of the coordination mechanism revising the essential package of health services, Somalia, 2020

In January 2020, early in the process of revising the package, the federal health ministry established a three-tier coordination structure and invited key stakeholders to nominate representatives for each level of the coordination structure (Fig. 1). Stakeholders chose their representatives based on technical expertise and knowledge of the health sector in Somalia. The ministry distributed a concept note to invited stakeholders, describing the scope and purpose of the revision process of the essential package. The note also contained a timeline and the expected contribution from stakeholders to the different stages of the planning cycle, such as evidence generation (data collection and analysis), priority setting, implementation strategy, and monitoring and evaluation.

The final coordination structure included representatives from the health ministry at the federal and state levels, finance ministry, civil society organizations, private sector, academia and development partners. These partners were: World Bank; Global Financing Facility; Foreign, Commonwealth and Development Office of the United Kingdom of Great Britain and Northern Ireland; World Health Organization; United Nations Children’s Fund; United Nations Population Fund; the Canadian, German, Italian and Swedish embassies; United States Agency for International Development; the Global Fund to Fight AIDS, Tuberculosis and Malaria; and Gavi, the Vaccine Alliance.

During the revision process, the health sector coordination committee, which includes all stakeholders, met on a quarterly basis, while the taskforce for the essential package of health services met every second month. The thematic technical working groups consisting of experts on different health system areas met more frequently. The majority of the meetings took place in a hybrid format, in person and virtually, due to the disruption caused by the coronavirus disease 2019 (COVID-19) pandemic. 

The government encouraged all stakeholders to harmonize and align their support with national priorities by using the single treasury account of the government to capture all development assistance for health in the national budget, consistent with the Paris Declaration on Aid Effectiveness of 2005 and the Accra Agenda for Action Plan of 2008, designed to enhance government ownership, efficiency for results and mutual accountability.^9^

## Relevant changes

Because of the constrained financial resources, and considering the limited delivery capacity of the health system, the taskforce had to make tough choices on what set of interventions in the package should be prioritized and rolled-out first across the five delivery levels of the health-care system of Somalia. The revision process entailed inclusive and in-depth discussions with all stakeholders, culminating in an agreement on the implementation sequence of interventions in the service package. The health ministry and development partners agreed upon the following criteria: (i) services that can address major causes associated with high mortality and morbidity in Somalia and have the most significant impact on health outcomes; (ii) highly cost-effective and affordable services within the available resources; (iii) services that can be scaled up to ensure equal access for all populations, regardless of residency. These criteria guided the prioritization and the selection of high-impact, cost-effective services which will be progressively realized to improve access and coverage, by considering the health system and operational capacity of service providers, particularly in security-compromised areas. 

After the revision process was finalized in June 2021, the health ministry and development partners started the progressive implementation by using a rational sequencing of health services included in the package. The outcomes of this implementation strategy have informed the long-term organizational transformation embarked upon by the health ministry. This transformation addresses institutional capacity, enhances domestic resources, and improves the availability of human resources, starting with the training of skilled community health workers in a gradual manner.

## Lessons learnt

The revision of the Essential Package of Health Services 2020^10^ benefitted from broad consultation and agreement with key stakeholders, whose contributions played a pivotal role in shaping the essential package and ensuring its alignment with the country's health goals and development priorities. This accomplishment was achieved through early engagement of stakeholders, by actively soliciting, analysing and incorporating their contributions and feedback into the essential package of health services. The revision process exemplifies the power of building consensus and fostering collaboration among stakeholders, aligned with the progress towards UHC. With a clear recognition of financial limitations and implementation capacity, the government and the development partners embraced a progressive rollout of the package and a pragmatic approach, prioritizing high-impact and cost-effective interventions within the essential package. This implementation strategy facilitated the progressive expansion of essential health services, tailored to address the country's diverse needs, service delivery capacity and resource availability (Box 1).

Box 1 **Summary of main lessons learnt**

- The development of an evidence-informed essential health services package was strengthened by effective stakeholder engagement, promoting strong government ownership and stewardship, and gaining support from donors and other stakeholders.
- Building consensus and collaboration among stakeholders requires a deliberative process, in which consistent evidence for necessary actions can build stakeholder trust and create opportunities for potential resource pooling to finance the service package.
- A unified approach, aimed at addressing the fragmentation issues that previously hindered the implementation of the essential package of health services, prepares for a more integrated and effective health-care delivery system.

However, the revision process faced challenges due to different positions held by some major stakeholders regarding the prioritization and financing of services, coverage for specific population groups and adopting a delivery model. Given the few available resources, these different positions necessitated an intensive policy dialogue to build consensus on balancing the breadth of coverage and services within the essential package of health services. As a result, the essential package of health services revision process took 18 months to finalize.

The service package, which defined a set of highly cost-effective interventions developed through collaborative endeavours, can strengthen the governance role and institutional capacity of the health ministry. A clear vision and roadmap towards UHC were foundational for the health ministry’s leadership. This vision, coupled with strong commitment and advance preparation, set the stage for a major revision involving stakeholders. Additionally, the adoption of the revised essential package as the national framework has been crucial for organizing, managing, and expanding health services, integrating across all programmes. As a result of these efforts, the health ministry has been able to lead and coordinate efforts in advancing health care in the country.

Unifying stakeholders is imperative to overcome the inefficiencies, fragmentation and unpredictability of funding. A collective approach towards funding and resource mobilization can be established by working together, leading to better coordination of funding sources. This collaboration enhances the predictability and sustainability of financing for the health-care system in Somalia, ensuring a stable foundation for the successful implementation of the essential package of health services.

Similarly, addressing the shortage of human resources necessitates stakeholder cooperation. Comprehensive workforce strategies can be devised through joint efforts, encompassing training programmes and incentives to attract and retain skilled health-care professionals. This collaborative approach will help bridge the health-care workforce gap, ensuring adequate staffing in health facilities.

The financing and the implementation of a nationally standardized package, which defined a set of health services to be delivered in Somalia under supervision and monitoring by federal and state health ministries, is a major departure from past experience where service providers could choose the parts of the package they preferred. The collective endeavour ensured buy-in from stakeholders, which eased the rollout of the revised package, even amidst resource constraints, and has advanced the health-care landscape in Somalia. Astute resource management by the health ministry and careful consideration of the health system's capacity addressed the financial challenges. Focusing on financing from available domestic and external resources, stakeholders have prioritized health services for maternal and child health, communicable diseases, and hypertension and diabetes, which needed improved public accessibility. The dedication to extending equitable access to nomadic and security-compromised areas of the country underscores a solid commitment to enhancing health-care accessibility and equity for all Somalis.
